# Impact of early ablation of atrial fibrillation on long-term outcomes: results from phase II/III of the GLORIA-AF registry

**DOI:** 10.1007/s00392-022-02022-1

**Published:** 2022-04-29

**Authors:** Wern Yew Ding, Peter Calvert, Dhiraj Gupta, Menno V. Huisman, Gregory Y. H. Lip, the GLORIA-AF Investigators

**Affiliations:** 1 Liverpool Centre for Cardiovascular Science, University of Liverpool and Liverpool Heart & Chest Hospital, Liverpool, UK; 2 Department of Thrombosis and Hemostasis, Leiden University Medical Center, Leiden, The Netherlands; 3 Aalborg Thrombosis Research Unit, Department of Clinical Medicine, Aalborg University, Aalborg, Denmark

**Keywords:** Early AF ablation, Long-term survival, Prognostic benefit, Newly diagnosed AF

## Abstract

**Background:**

First-line ablation for atrial fibrillation (AF) reduces the risk of recurrent atrial arrhythmias compared to medical therapy. However, the prognostic benefit of early AF ablation remains undetermined. Herein, we aimed to evaluate the effects of early AF ablation compared to medical therapy.

**Methods:**

Using data from phase II/III of the GLORIA-AF registry, we studied patients who were consecutively enrolled with newly diagnosed AF (< 3 months before baseline visit) and an increased risk of stroke (CHA_2_DS_2_–VASc ≥ 1). At baseline visit, 445 (1.7%) patients were treated with early AF ablation and 25,518 (98.3%) with medical therapy. Outcomes of interest were the composite outcome of all-cause death, stroke and major bleeding, and pre-specified outcomes of all-cause death, cardiovascular (CV) death, non-CV death, stroke and major bleeding.

**Results:**

A total of 25,963 patients (11733 [45.2%] females; median age 71 [IQR 64–78] years; 17424 [67.1%] taking non-vitamin K antagonist oral anticoagulants [NOACs]) were included. Over a follow-up period of 3.0 (IQR 2.3–3.1) years, after adjustment for confounders, early AF ablation was associated with a significant reduction in the composite outcome of all-cause death, stroke and major bleeding (HR 0.50 [95% CI 0.30–0.85]) and all-cause death (HR 0.45 [95% CI 0.23–0.91]). There were no statistical differences between the groups in terms of CV death, non-CV death, stroke and major bleeding. Similar results were obtained in a propensity-score matched analysis of patients with comparable baseline variables.

**Conclusions:**

Early AF ablation in a contemporary prospective cohort of AF patients who were predominantly treated with NOACs was associated with a survival advantage compared to medical therapy alone.

**Trial registration:**

Clinical trial registration: http://www.clinicaltrials.gov. Unique identifiers: NCT01468701, NCT01671007 and NCT01937377.

**Graphical abstract:**

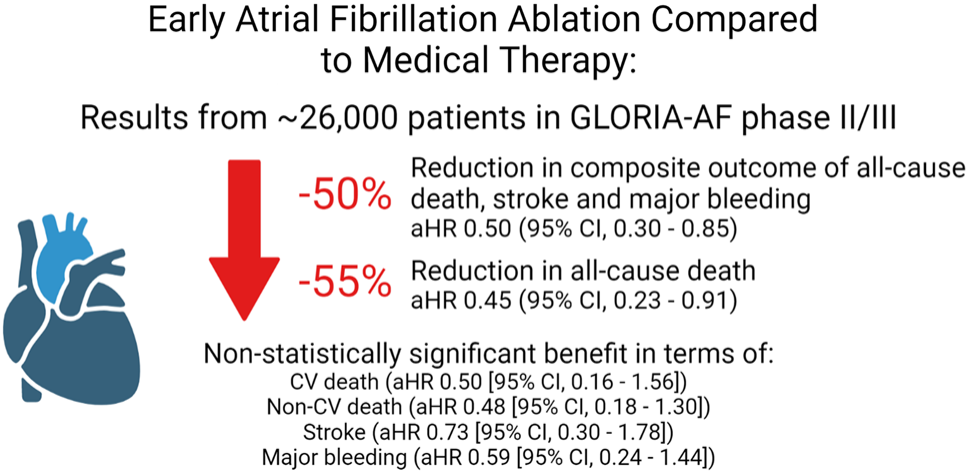

Created with BioRender.com.

**Supplementary Information:**

The online version contains supplementary material available at 10.1007/s00392-022-02022-1.

## Introduction

Atrial fibrillation (AF) is a cardiac arrhythmia that is characterised by an irregular heart beat and has important interactions with numerous other conditions. It is associated with an increased risk of thromboembolic complications, heart failure and death [1–3], and excess healthcare costs [4]. The treatment of patients with AF includes the use of either rhythm or rate control management strategies to achieve symptom control, as per current international guidelines [5–7]. These strategies were accepted as equivocal on the basis of historical studies which demonstrated similar outcomes with both [8]. Nonetheless, this notion has been challenged by more recent evidence from the EAST–AFNET 4 (Early Treatment of Atrial Fibrillation for Stroke Prevention) trial suggesting that early rhythm control may offer a prognostic advantage over rate control [9].

Rhythm control may be achieved using either cardioversion with anti-arrhythmic drugs or electrical therapy, or AF ablation. Over the past decade, the superiority of AF ablation over anti-arrhythmic drugs for the prevention of atrial arrhythmia recurrence has been proven [10], such that there is now an increasing argument for its use as first-line treatment in patients with AF. Recently, we published a systematic review and meta-analysis of 6 randomised controlled trials demonstrating that first-line treatment with catheter AF ablation was associated with a 36% reduction in the recurrence of atrial arrhythmias and 47% reduction in healthcare resource utilisation compared to anti-arrhythmic drug therapy [11].

Despite the advantages of AF ablation as described above, its benefits among the general AF population in terms of hard clinical outcomes including stroke, heart failure and long-term survival remain ill-defined. Several observational studies previously reported a prognostic benefit with AF ablation [12–15], though this was not found in the CABANA (Catheter Ablation vs. Antiarrhythmic Drug Therapy for Atrial Fibrillation) randomised controlled trial [10]. Nonetheless, the aforementioned studies were not designed to test for the effects of early AF ablation and only 8% of patients in the early rhythm control arm of EAST–AFNET 4 received such treatment [9]. Herein, we aimed to evaluate the effects of early AF ablation in patients from the contemporary prospective GLORIA-AF (Global Registry on Long-Term Oral Anti-thrombotic Treatment In Patients With Atrial Fibrillation) registry.

## Methods

### Study design and population

GLORIA-AF is a prospective, observational, global registry programme of patients from 935 centres across 38 participating countries in Asia, Europe, North America, Latin America, and Africa/Middle East. The study design has previously been described [16]. In brief, consecutive adults with newly diagnosed AF (< 3 months before baseline visit) and an increased risk of stroke (CHA_2_DS_2_–VASc ≥ 1) were enrolled. This study focused on patients from GLORIA-AF phase II and III. These patients were enrolled between 2011 and 2020. Patients with known ablation status at baseline and follow-up data were included. Main exclusion criteria of GLORIA-AF registry were the presence of mechanical heart valve or valvular disease necessitating valve replacement, prior oral anticoagulation with vitamin K oral antagonist over 60 days, a reversible cause of AF, indication for anticoagulation other than AF, and life expectancy of less than 1 year. Ethics approval was obtained from the local institutional review boards, informed consent was obtained from patients, and the study was performed in accordance with the Declaration of Helsinki.

### Data collection and definition

Data on demographics, comorbidities and therapies were collected at baseline with standardised, prospectively designed data collection tools. Early AF ablation was defined as AF ablation within 3 months from diagnosis. Creatinine clearance (CrCl) was assessed using the Cockcroft–Gault equation [17]. AF classification was determined according to the European Society of Cardiology recommendations [18]. Severity of AF-related symptoms was ascertained using the European Heart Rhythm Association classification [19]. CHADS_2_, CHA_2_DS_2_–VASc and HAS–BLED scores were calculated as previously described [20–22].

### Study outcomes and follow-up

Outcomes of interest were the composite outcome of all-cause death, stroke and major bleeding, and the pre-specified outcomes of all-cause death, cardiovascular (CV) death, non-CV death, stroke and major bleeding. Stroke was defined as an acute onset of a focal neurological deficit of presumed vascular origin lasting for 24 h or more, or resulting in death. Major bleeding was defined as either overt bleeding associated with a reduction in haemoglobin of at least 20 g/L or leading to a transfusion of at least 2 units of blood or packed cells; symptomatic bleeding in a critical area or organ (intraocular, intracranial, intraspinal or intramuscular with compartment syndrome, retroperitoneal bleeding, intra-articular bleeding or pericardial bleeding); or life-threatening bleeding. In phase II, follow-up for the dabigatran cohort was for 2 years, with scheduled visits at 3, 6, 12, and 24 months. In phase III, follow-up for all patients was conducted for 3 years, with scheduled visits at 6, 12, 24, and 36 months.

### Statistical analysis

Continuous variables were described with median and interquartile range (IQR), and tested for differences with Kruskal–Wallis test. Categorical variables were described as count and percentage, and tested for differences with chi-squared test. Plots of Kaplan–Meier curves were performed for each outcome and survival distributions were compared using log-rank test. Cox proportional hazards analyses were performed to study the effects of early AF ablation on outcomes of interest. Potential confounders were accounted for using a multivariable model with forward selection of covariates including age, gender, body mass index, CrCl, type of AF, hypertension, hyperlipidaemia, diabetes mellitus, coronary artery disease, heart failure, left ventricular hypertrophy, prior thromboembolism, prior bleeding, peripheral artery disease, chronic obstructive pulmonary disease, oral anticoagulation use, antiplatelet use, anti-arrhythmic drug therapy, angiotensin-converting enzyme inhibitor, angiotensin receptor blocker, beta-blocker, digoxin, statin and diuretic therapy.

Further analyses were undertaken after rigorous adjustment for baseline characteristics with propensity score matching (PSM) generated by logistic regression for all variables in Supplementary Table 1 in a 1:1 ratio using the nearest-neighbour technique without replacement. A two-sided *p* value of less than 0.05 was considered statistically significant. Statistical analyses were performed using RStudio (Version 1.3.1093).

## Results

**Fig. 1:**
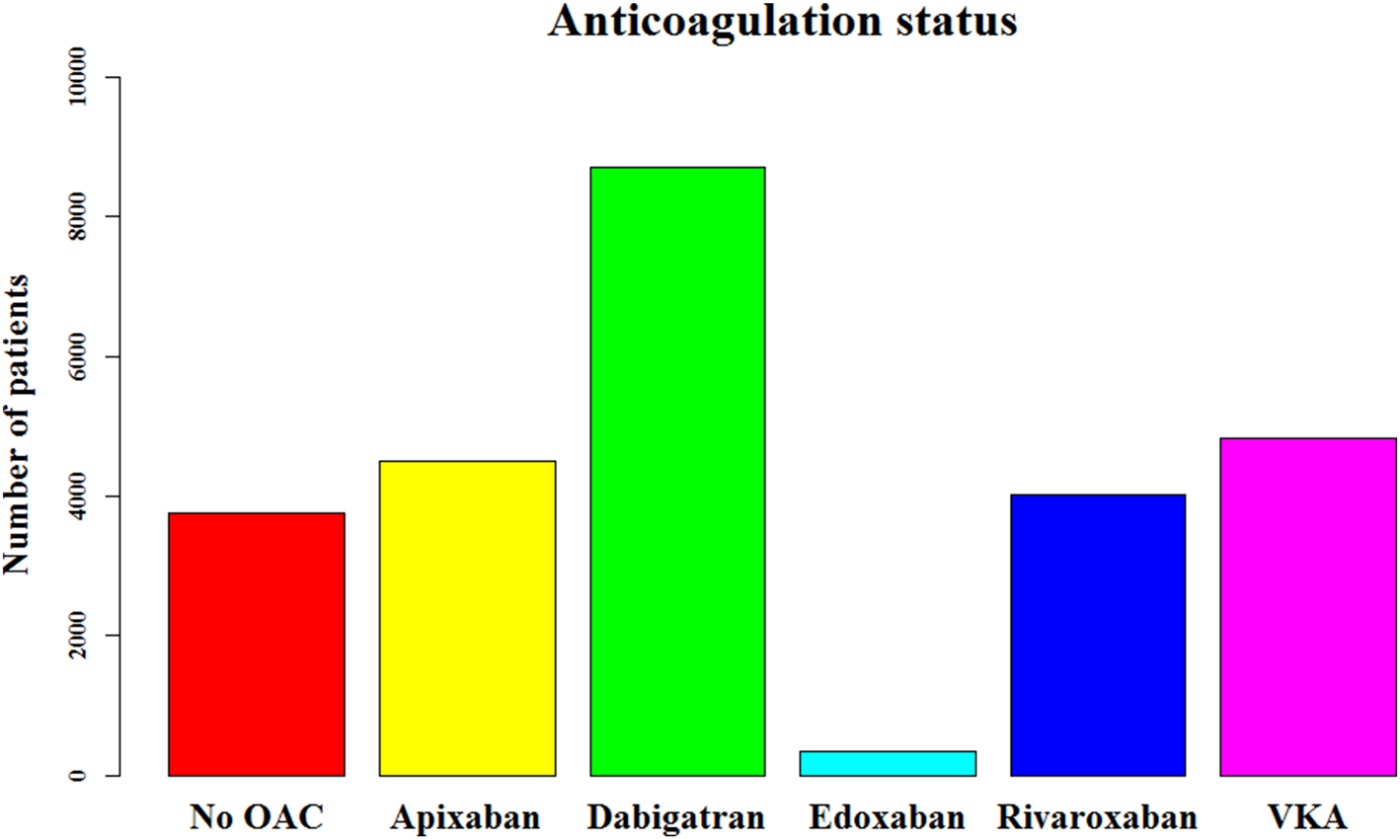
Anticoagulation status of patients in study cohort (*n* = 25,963). *OAC* oral anticoagulant, *VKA* vitamin K antagonist

A total of 25,963 patients (11,733 [45.2%] females; median age 71 [IQR 64–78] years) were included. A flow chart of the patient selection process is shown in Supplementary Fig. 1. The anticoagulation status of patients in this study cohort is demonstrated in Fig. 1. There were 445 (1.7%) patients treated with early AF ablation and 25,518 (98.3%) with medical therapy.

### Baseline characteristics

Baseline characteristics are described in Table 1. Patients in the early AF ablation group were younger and had lower body mass index, higher CrCl, less advanced forms of AF and reduced burden of comorbidities including hypertension, hypercholesterolaemia, diabetes mellitus, prior myocardial infarction, heart failure, left ventricular hypertrophy, prior thromboembolism and chronic obstructive pulmonary disease compared to patients on medical therapy alone. As a result, patients treated with early AF ablation had a lower risk of stroke (median CHADS_2_ of 1 [IQR 1–2] vs. 2 [IQR 1–3]; CHA_2_DS_2_–VASc of 2 [IQR 1–3] vs. 3 [IQR 2–4]) and major bleeding (median HAS–BLED of 1 [IQR 0–1] vs. 1 [IQR 1–2]).

**Table 1:** Baseline characteristics

Baseline characteristics	Early AF ablation (*n* = 445)	Medical therapy (*n* = 25,518)	*p* value
Age (years), median (IQR)	63 (57–70)	71 (64–78)	< 0.001
Female sex, *n* (%)	186 (41.8%)	11,450 (44.9%)	0.213
Heart rate (bpm), median (IQR)	75 (67–86)	76 (65–90)	0.394
sBP (mmHg), median (IQR)	130 (120–140)	130 (120–142)	0.003
BMI (kg/m^2^), median (IQR)	26.1 (23.6–29.1)	27.6 (24.5–31.5)	< 0.001
CrCl (mL/min), median (IQR)	84.7 (68.6–107.0)	75.3 (56.9–98.3)	< 0.001
AF classification, *n* (%)			
Paroxysmal	332 (74.6%)	14,084 (55.2%)	< 0.001
Persistent	110 (24.7%)	8807 (34.5%)	
Permanent	3 (0.7%)	2627 (10.3%)	
EHRA classification, *n* (%)			
I	45 (10.7%)	8732 (36.2%)	< 0.001
II	177 (42.1%)	9027 (37.4%)	
III	179 (42.6%)	4906 (20.3%)	
IV	19 (4.5%)	1471 (6.1%)	
Comorbidities, *n* (%)			
Hypertension	308 (69.2%)	19,218 (75.5%)	0.003
Hypercholesterolaemia	125 (28.3%)	10,171 (40.9%)	< 0.001
Diabetes mellitus	82 (18.4%)	5937 (23.3%)	0.019
Coronary artery disease	85 (19.3%)	4810 (19.3%)	1.000
Prior myocardial infarction	12 (2.7%)	2457 (9.6%)	< 0.001
Heart failure	63 (14.2%)	5708 (22.5%)	< 0.001
Left ventricular hypertrophy	51 (11.6%)	4735 (19.4%)	< 0.001
Prior thromboembolism	46 (10.3%)	3805 (14.9%)	0.009
Prior stroke	36 (8.1%)	2783 (10.9%)	0.069
Prior bleeding	17 (3.9%)	1356 (5.4%)	0.188
Peripheral artery disease	7 (1.6%)	744 (2.9%)	0.120
COPD	14 (3.2%)	1546 (6.1%)	0.013
CHADS_2_ score, median (IQR)	1 (1–2)	2 (1–3)	< 0.001
CHA_2_DS_2_–VASc score, median (IQR)	2 (1–3)	3 (2–4)	< 0.001
HAS–BLED score, median (IQR)	1 (0–1)	1 (1–2)	< 0.001

*AF* atrial fibrillation, *BMI* body mass index, *COPD* chronic obstructive pulmonary disease, *CrCl* creatinine clearance, *EHRA* European heart rhythm association, *IQR* interquartile range, *sBP* systolic blood pressure

### Medication use

Oral anticoagulation was prescribed in 22,219 (85.6%) patients at baseline with 17,424 (67.1%) receiving non-vitamin K antagonist oral anticoagulants and 4795 (18.5%) vitamin K antagonist. In contrast to patients on medical therapy, those treated with early AF ablation had greater uptake of anticoagulation and anti-arrhythmic drug therapy but less frequent use of antiplatelet agent, angiotensin-converting enzyme inhibitor, beta-blocker, digoxin and diuretic therapy (Table 2).

**Table 2:** Medication use

Medication use	Early AF ablation (*n* = 445)	Medical therapy (*n* = 25,518)	*p* value
Anticoagulation, *n* (%)	402 (90.5%)	21,817 (85.5%)	0.004
Apixaban	39 (8.8%)	4429 (17.4%)	
Dabigatran	216 (48.6%)	8427 (33.0%)	
Edoxaban	7 (1.6%)	323 (1.3%)	
Rivaroxaban	53 (11.9%)	3930 (15.4%)	
VKA	87 (19.6%)	4708 (18.5%)	
Antiplatelet, *n* (%)	68 (15.3%)	6223 (24.4%)	< 0.001
Anti-arrhythmic drug, *n* (%)	215 (48.3%)	6488 (25.4%)	< 0.001
ACE-i, *n* (%)	95 (21.3%)	7814 (30.6%)	< 0.001
Angiotensin receptor blocker, *n* (%)	104 (23.4%)	6572 (25.8%)	0.278
Beta-blocker, *n* (%)	182 (40.9%)	16,159 (63.3%)	< 0.001
Digoxin, *n* (%)	13 (2.9%)	2135 (8.4%)	< 0.001
Diuretic, *n* (%)	81 (18.2%)	9770 (38.3%)	< 0.001
Statin, *n* (%)	179 (40.2%)	11,475 (45.0%)	0.052

*AF* atrial fibrillation, *ACE*-*i* angiotensin-converting enzyme inhibitor, *VKA* vitamin K antagonist

### Study outcomes

**Fig. 2:**
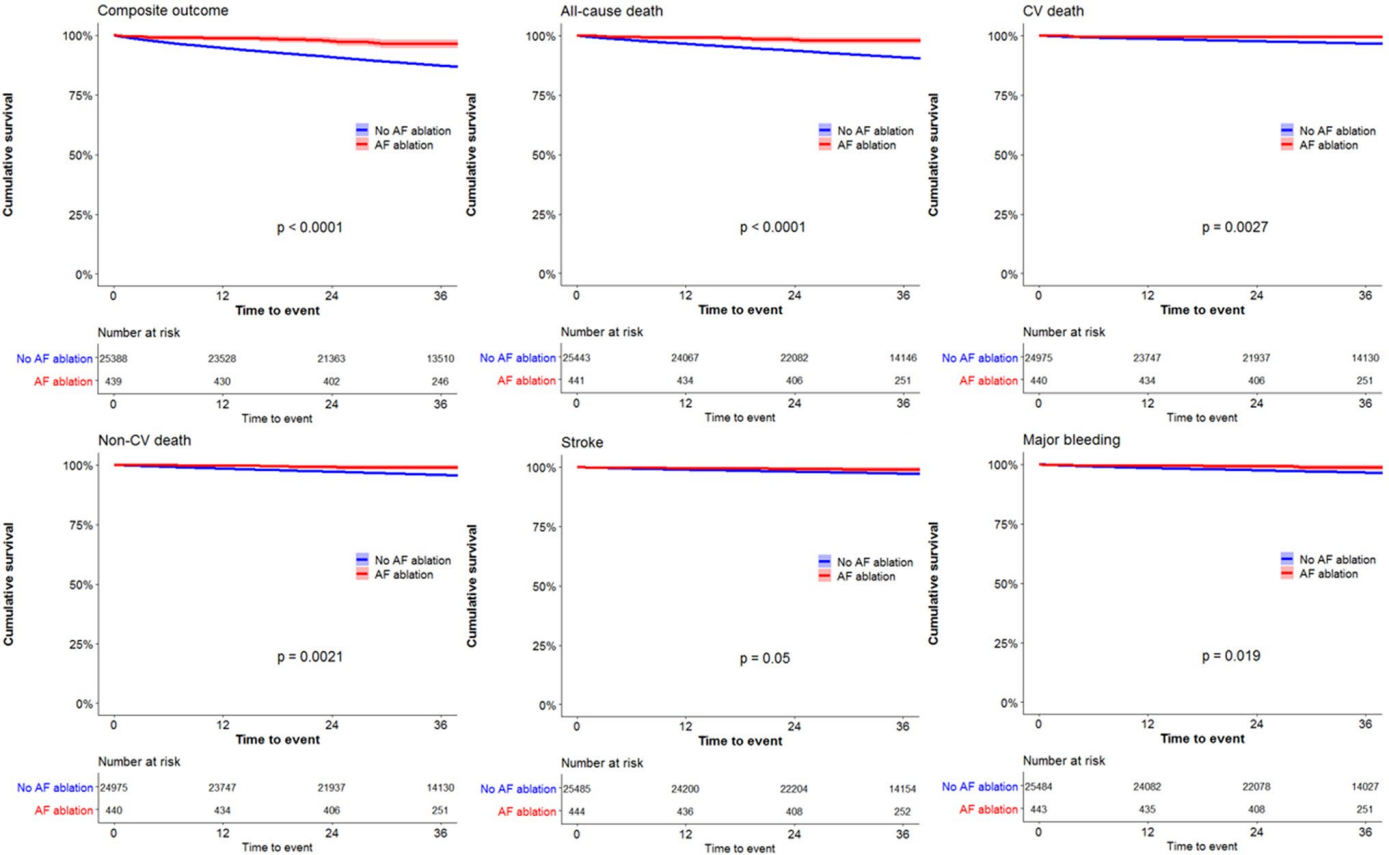
Kaplan–Meier survival curves for composite outcome of all-cause death, stroke and major bleeding, all-cause death, CV death, non-CV death, stroke and major bleeding

During a median follow-up period of 3.0 (IQR 2.3–3.1) years, there were 3237 (12.5%) events for the composite outcome of all-cause death, stroke and major bleeding, including 2305 (8.9%) all-cause deaths, 807 (3.1%) CV deaths, 989 (3.8%) non-CV deaths, 679 (2.6%) strokes and 870 (3.4%) major bleeding events. Kaplan–Meier survival analyses demonstrated that patients who were treated with early AF ablation had lower event rates for the composite outcome, all-cause death, CV death, non-CV death and major bleeding with a trend for reduced stroke compared to patients on medical therapy alone (Fig. 2).

Early AF ablation was associated with a significant reduction in the composite outcome of all-cause death, stroke and major bleeding (HR 0.26 [95% CI, 0.16–0.43]), all-cause death (HR 0.22 [95% CI, 0.11–0.42]), CV death (HR 0.21 [95% CI, 0.07–0.65]), non-CV death (HR 0.28 [95% CI, 0.11–0.66]), stroke (HR 0.43 [95% CI, 0.18–1.00]) and major bleeding (HR 0.39 [95% CI, 0.18–0.88]) (Table 3). After adjustment for various confounders, the composite outcome (HR 0.50 [95% CI, 0.30–0.85]) and all-cause death (HR 0.45 [95% CI, 0.23–0.91]) remained significantly lower among patients who were treated with early AF ablation compared to those on medical therapy.

**Table 3:** Effects of early AF ablation vs. medical therapy

Outcomes	*n* (%)		Early AF ablation vs. medical therapy		
	Early AF ablation (*n* = 445)	Medical therapy (*n* = 25,518)	Univariate HR (95% CI)	aHR^†^ (95%)	*p* value
Composite outcome of all-cause death, stroke and major bleeding	21 (4.7%)	3216 (12.6%)	0.26 (0.16–0.43)	0.50 (0.30–0.85)	0.011
All-cause death	13 (2.9%)	2292 (9.0%)	0.22 (0.11–0.42)	0.45 (0.23–0.91)	0.027
CV death	5 (1.1%)	802 (3.2%)	0.21 (0.07–0.65)	0.50 (0.16–1.56)	0.232
Non-CV death	6 (1.4%)	983 (3.9%)	0.28 (0.11–0.66)	0.48 (0.18–1.30)	0.149
Stroke	6 (1.4%)	673 (2.6%)	0.43 (0.18–1.00)	0.73 (0.30–1.78)	0.489
Major bleeding	8 (1.8%)	862 (3.4%)	0.39 (0.18–0.88)	0.59 (0.24–1.44)	0.247

*AF* atrial fibrillation, *aHR* adjusted hazard ratio, *CI* confidence interval, *CV* cardiovascular, *HR* hazard ratio

^†^Adjusted for age, gender, body mass index, creatinine clearance, type of atrial fibrillation, hypertension, hyperlipidaemia, diabetes mellitus, coronary artery disease, heart failure, left ventricular hypertrophy, prior thromboembolism, prior bleeding, peripheral artery disease, chronic obstructive pulmonary disease, oral anticoagulation use, antiplatelet use, anti-arrhythmic drug therapy, angiotensin-converting enzyme inhibitor, angiotensin receptor blocker, beta-blocker, digoxin, statin and diuretic therapy

**Fig. 3:**
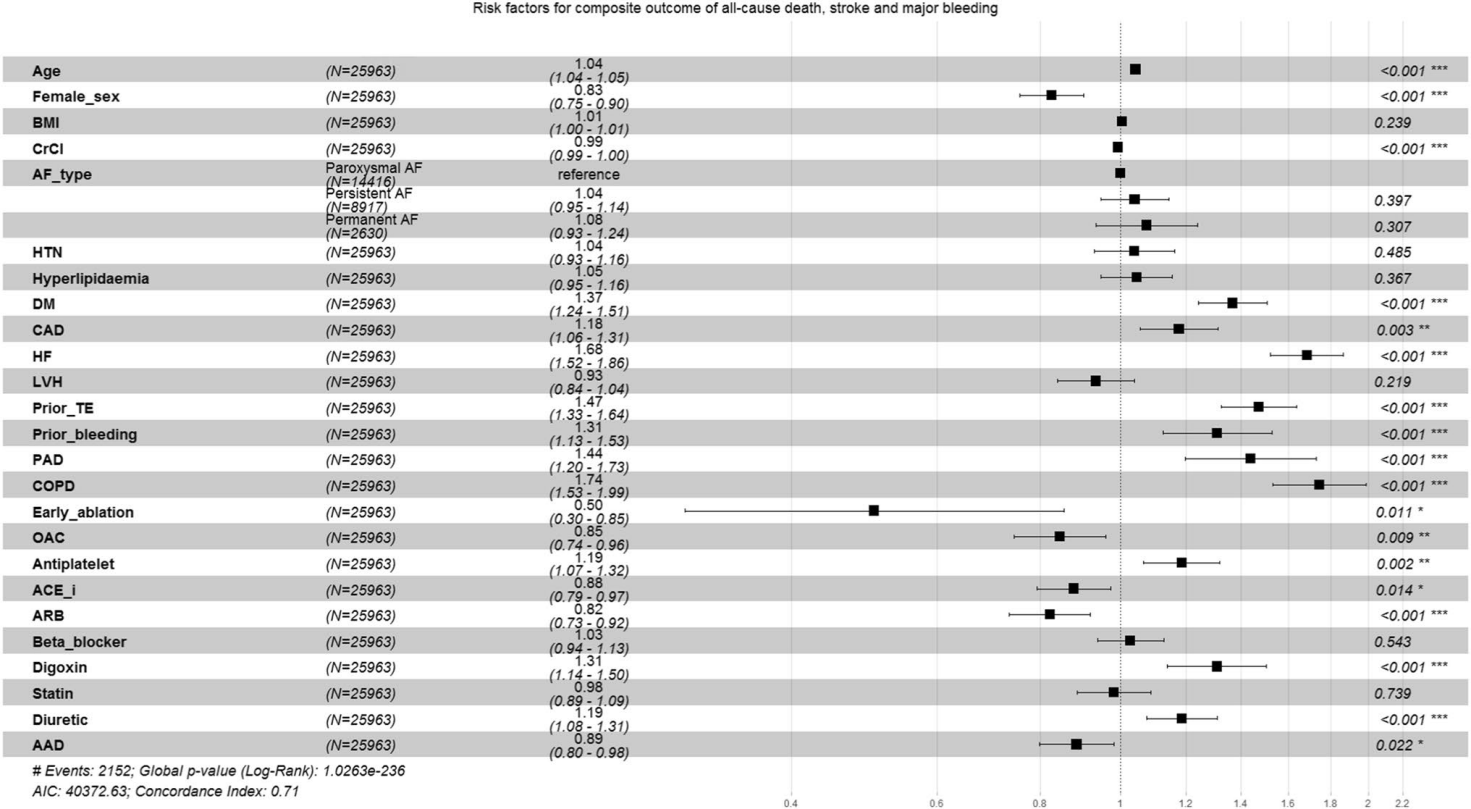
Effects of early AF ablation in comparison to other risk factors for the composite outcome of all-cause death, stroke and major bleeding. *AAD* anti-arrhythmic drug, *ACE*-*i* angiotensin-converting enzyme inhibitor, *AF* atrial fibrillation, *ARB* angiotensin receptor blocker, *BMI* body mass index, *CAD* coronary artery disease, *COPD* chronic obstructive pulmonary disease, *CrCl* creatinine clearance, *DM* diabetes mellitus, *HF* heart failure, *HTN* hypertension, *LVH* left ventricular hypertrophy, *OAC* oral anticoagulation, *PAD* peripheral artery disease, *TE* thromboembolism

A comparison of early AF ablation to other risk factors for the composite outcome of all-cause death, stroke and major bleeding are shown in Fig. 3. Worse outcomes were seen in patients with increased age, reduced renal function, diabetes mellitus, coronary artery disease, heart failure, prior thromboembolism, prior bleeding, peripheral artery disease, chronic obstructive pulmonary disease, antiplatelet use, digoxin and diuretic therapy. Protective factors were early AF ablation, female sex, oral anticoagulation use, angiotensin-converting enzyme inhibitor, angiotensin receptor blocker and anti-arrhythmic drug therapy.

### PSM cohort

Using PSM, we identified 399 patients in each group with comparable baseline characteristics (Supplementary Table 1). Within this cohort, early AF ablation was related to a decrease in the composite outcome of all-cause death, stroke and major bleeding (HR 0.41 [95% CI, 0.22–0.77]) and all-cause death (HR 0.43 [95% CI, 0.19–0.99]) (Table 4). There was no statistically significant difference between the groups in terms of CV death, non-CV death, stroke and major bleeding.

**Table 4:** Effects of early AF ablation vs. medical therapy *after* propensity score matching

Outcomes	*n* (%)		HR (95% CI)	*p* value
	Early AF ablation (*n* = 399)	Medical therapy (*n* = 399)		
Composite outcome of all-cause death, stroke and major bleeding	18 (4.5%)	33 (8.3%)	0.41 (0.22–0.77)	0.006
All-cause death	10 (2.5%)	18 (4.5%)	0.43 (0.19–0.99)	0.046
CV death	4 (1.0%)	8 (2.0%)	0.36 (0.10–1.40)	0.130
Non-CV death	5 (1.3%)	9 (2.3%)	0.43 (0.13–1.40)	0.160
Stroke	5 (1.3%)	9 (2.3%)	0.60 (0.20–1.80)	0.370
Major bleeding	7 (1.8%)	11 (2.8%)	0.42 (0.15–1.20)	0.110

*AF* atrial fibrillation, *CI* confidence interval, *CV* cardiovascular, *HR* hazard ratio

## Discussion

In this large, global, prospective registry of patients with newly diagnosed AF who were predominantly treated with non-vitamin K antagonist oral anticoagulants, we present novel findings demonstrating that early AF ablation was independently associated with a 51% decrease in the composite outcome of all-cause death, stroke and major bleeding, and a 59% decrease in all-cause death compared to medical therapy alone over a 3-year follow-up period. Second, early AF ablation had the greatest benefit in terms of reducing the composite outcome vs. other therapies, such as oral anticoagulation; however, less than 2% of all patients with newly diagnosed AF received such treatment in this cohort.

The benefits of ablation therapy among patients with AF have previously been reported in observational studies across different centres and countries. A retrospective study using MarketScan administrative claims data showed that patients who were treated with AF ablation had a reduced risk of thromboembolic events compared to those on anti-arrhythmic drugs [12]. Another large population-based study from the United States found that patients with AF who were treated with an ablation procedure had significantly lower all-cause death, ischaemic stroke and haemorrhagic stroke compared to matched controls [13]. A propensity score-matched population-based study from Israel of patients with AF and predominantly a high CHA_2_DS_2_–VASc score found that catheter ablation was associated with a decreased risk of stroke or transient ischaemic attack, and all-cause death [14]. Another recent study using the Korean National Health Insurance database demonstrated that catheter AF ablation compared to medical therapy significantly reduced the risk of both ischaemic stroke and intracranial haemorrhage [23]. Furthermore, patients treated with catheter AF ablation had similar outcomes as the non-AF population. However, all of these retrospective studies suffer from potential selection bias which may have influenced the results in favour of AF ablation [24].

In contrast, the CABANA randomised trial found that there was no significant difference in the primary composite outcome of death, disabling stroke, serious bleeding or cardiac arrest between catheter ablation and medical therapy in patients with AF [10]. This trial was, however, plagued by several issues including cross-over of a considerable number of patients from medical therapy to catheter AF ablation, significant loss to follow-up or withdrawal, and lower than expected event rates which may have substantially compromised the validity of the results. Interestingly, pre-specified as-treated analyses demonstrated that the primary composite outcome was reduced with catheter AF ablation, as was the risk of death [10].

Given the differences in findings between observational studies and randomised controlled trials, it is perhaps unsurprising that there were conflicting results between meta-analyses on this topic, depending on the study selection criteria. In a meta-analysis of 11 randomised controlled trials comprised of 1763 patients, catheter AF ablation compared to anti-arrhythmic drug therapy led to a reduction in AF recurrence and improvement in quality of life but failed to alter the risk of all-cause death and stroke or transient ischaemic attack [25]. Another meta-analysis of 13 randomised controlled trials comprised of 3856 patients without heart failure also reported no significant difference between catheter AF ablation and medical therapy in terms of all-cause death and stroke [26]. However, patients who received catheter AF ablation had reduced cardiac hospitalisation and less frequent atrial arrhythmia recurrence, at the expense of procedural complications, such as pericardial tamponade; highlighting the need to recognise the invasive nature of AF ablation and to balance any intended benefits with the risk of potential complications [27]. Conversely, a meta-analysis of 9 studies (8 matched population studies and 1 randomised controlled trial) with 241,372 patients with AF found that catheter ablation decreased the risk of death, stroke and hospitalisation for heart failure compared to medical therapy alone [28].

Unlike the aforementioned studies, we investigated the effects of early AF ablation (i.e., within 3 months from diagnosis) in a prospectively enrolled global AF population with a high uptake of oral anticoagulation and found a significant survival benefit in these patients compared to those who received medical therapy alone. While we found a reduction in the risk of all-cause death with early AF ablation, we were unable to attribute this to CV or non-CV death likely owing to the low event rates. Notably, only a minority of patients were treated with early AF ablation and there were significant differences between the groups at baseline. Nonetheless, our results provide further support for the emerging role of early AF ablation and complements existing evidence from the EAST-AFNET 4 trial [9].

Current recommendations from international guidelines advocate that AF ablation should be reserved for patients who have failed at least 1 anti-arrhythmic drug therapy, though it may be considered in selected patients with early forms of AF or heart failure with reduced ejection fraction [5–7]. Healthcare structures in most countries are such that there is often a delay between the diagnosis of AF and specialist review, and/or subsequent referral for consideration of AF ablation. As a result, adoption of early AF ablation is uncommon, as observed in the present study. However, there is growing evidence to suggest that AF ablation should be considered early in the patient journey, consistent with the understanding that atrial remodelling occurs with chronic disease and hence the phrase ‘AF begets AF’ [29]. Furthermore, the importance of maintaining sinus rhythm should not be underestimated [30] and the success of catheter AF ablation is reduced with treatment delay [31]. To this end, systematic improvements are needed to facilitate the delivery of early AF ablation to patients who are eligible and may benefit from such treatment. Such facilitation is an argument for a more integrated care approach to patient care pathways, including for those AF and other chronic cardiovascular conditions [32, 33]. Indeed, adherence to a holistic or integrated care approach to AF care is associated with improved clinical outcomes [34, 35].

### Limitations

The main limitations of this study are linked to possible misclassification and selection bias due its observational nature; though consecutive enrolment of patients was performed to reduce selection bias. As only a small proportion of patients were treated with early AF ablation, the results of this highly selected group may not be generalisable to the wider AF population. Despite rigorous model adjustment and propensity matching to ensure a balance of comorbidities and medication use between the groups, some residual unmeasured confounders may exist. Therefore, we are unable to prove a cause–effect relationship. In addition, outcomes were analysed according to variables collected at baseline. In this regard, a proportion of patients in the medical therapy group may have been treated with AF ablation following enrolment. This may have attenuated any differences between the groups but serves to provide further strength to our positive findings. Overall, the results of this post-hoc analysis should be treated as hypotheses-generating.

## Conclusions

Early AF ablation within 3 months from initial diagnosis in a contemporary cohort of patients who were predominantly treated with non-vitamin K antagonist oral anticoagulants was associated with a survival advantage compared to medical therapy alone. Moreover, early AF ablation appeared to provide the greatest benefit compared to other treatments.

## Supplementary Information

Below is the link to the electronic supplementary material.

Supplementary file1 (DOCX 371 KB)

Supplementary file2 (DOCX 38 KB)

## Data Availability

The data underlying this article will be shared on reasonable request to the corresponding author.

## References

[1] Benjamin EJ, Wolf PA, D’Agostino RB (1998). Impact of atrial fibrillation on the risk of death: the Framingham heart study. Circulation.

[2] Stewart S, Hart CL, Hole DJ, McMurray JJ (2002). A population-based study of the long-term risks associated with atrial fibrillation: 20-year follow-up of the Renfrew/Paisley study. Am J Med.

[3] Vermond RA, Geelhoed B, Verweij N (2015). Incidence of atrial fibrillation and relationship with cardiovascular events, heart failure, and mortality a community-based study from the Netherlands. J Am Coll Cardiol.

[4] Burdett P, Lip GYH (2020). Atrial fibrillation in the United Kingdom: predicting costs of an emerging epidemic recognising and forecasting the cost drivers of atrial fibrillation-related costs. Eur Hear J-Qual Care Clin Outcomes.

[5] Hindricks G, Potpara T, Dagres N (2021). 2020 ESC guidelines for the diagnosis and management of atrial fibrillation developed in collaboration with the European association of cardio-thoracic surgery (EACTS). Eur Heart J.

[6] January CT, Wann LS, Alpert JS (2014). 2014 AHA/ACC/HRS guideline for the management of patients with atrial fibrillation: executive summary: a report of the American college of cardiology/American heart association task force on practice guidelines and the heart rhythm society. Circulation.

[7] January CT, Wann LS, Calkins H (2019). 2019 AHA/ACC/HRS focused update of the 2014 AHA/ACC/HRS guideline for the management of patients with atrial fibrillation: a report of the American college of cardiology/American heart association task force on clinical practice guidelines and the heart R. J Am Coll Cardiol.

[8] Sethi NJ, Feinberg J, Nielsen EE (2017). The effects of rhythm control strategies versus rate control strategies for atrial fibrillation and atrial flutter: a systematic review with meta-analysis and trial sequential analysis. PLoS One.

[9] Kirchhof P, Camm AJ, Goette A (2020). Early rhythm-control therapy in patients with atrial fibrillation. N Engl J Med.

[10] Packer DL, Mark DB, Robb RA (2019). Effect of catheter ablation vs antiarrhythmic drug therapy on mortality, stroke, bleeding, and cardiac arrest among patients with atrial fibrillation: the CABANA randomized clinical trial. JAMA.

[11] Imberti JF, Ding WY, Kotalczyk A (2021). Catheter ablation as first-line treatment for paroxysmal atrial fibrillation: a systematic review and meta-analysis. Heart.

[12] Mansour M, Heist EK, Agarwal R (2018). Stroke and cardiovascular events after ablation or antiarrhythmic drugs for treatment of patients with atrial fibrillation. Am J Cardiol.

[13] Srivatsa UN, Danielsen B, Amsterdam EA (2018). CAABL-AF (California study of ablation for atrial fibrillation): mortality and stroke, 2005 to 2013. Circ Arrhythm Electrophysiol.

[14] Saliba W, Schliamser JE, Lavi I (2017). Catheter ablation of atrial fibrillation is associated with reduced risk of stroke and mortality: a propensity score-matched analysis. Hear Rhythm.

[15] Ding WY, Williams E, Das M (2021). Cryoballoon pulmonary vein isolation as first line treatment for typical atrial flutter (CRAFT): study protocol for a randomised controlled trial. J Interv Card Electrophysiol.

[16] Huisman MV, Lip GYH, Diener HC (2014). Design and rationale of global registry on long-term oral antithrombotic treatment in patients with atrial fibrillation: a global registry program on long-term oral antithrombotic treatment in patients with atrial fibrillation. Am Heart J.

[17] Cockcroft DW, Gault MH (1976). Prediction of creatinine clearance from serum creatinine. Nephron.

[18] Kirchhof P, Benussi S, Kotecha D (2016). 2016 ESC guidelines for the management of atrial fibrillation developed in collaboration with EACTS. Eur Heart J.

[19] Kirchhof P, Auricchio A, Bax J (2007). Outcome parameters for trials in atrial fibrillation: recommendations from a consensus conference organized by the German atrial fibrillation competence NETwork and the European heart rhythm association. Europace.

[20] Lip GYH, Nieuwlaat R, Pisters R (2010). Refining clinical risk stratification for predicting stroke and thromboembolism in atrial fibrillation using a novel risk factor-based approach: the Euro heart survey on atrial fibrillation. Chest.

[21] Pisters R, Lane DA, Nieuwlaat R (2010). A novel user-friendly score (HAS-BLED) to assess 1-year risk of major bleeding in patients with atrial fibrillation: the Euro heart survey. Chest.

[22] Gage BF, Waterman AD, Shannon W (2001). Validation of clinical classification schemes for predicting stroke: results from the National registry of atrial fibrillation. JAMA.

[23] Kim M, Yu HT, Kim J (2021). Atrial fibrillation and the risk of ischaemic strokes or intracranial haemorrhages: comparisons of the catheter ablation, medical therapy, and non-atrial fibrillation population. Europace.

[24] Ding WY, Gupta D (2021). Catheter ablation: the “Pym particles” of atrial fibrillation?. Europace.

[25] Shi L-Z, Heng R, Liu S-M, Leng F-Y (2015). Effect of catheter ablation versus antiarrhythmic drugs on atrial fibrillation: a meta-analysis of randomized controlled trials. Exp Ther Med.

[26] Muhammad ZK, Safi UK, Adeel A (2020). Meta-analysis of catheter ablation versus medical therapy in patients with atrial fibrillation without heart failure. J Atr Fibrillation.

[27] Gupta A, Perera T, Ganesan A (2013). Complications of catheter ablation of atrial fibrillation: a systematic review. Circ Arrhythm Electrophysiol.

[28] Saglietto A, De Ponti R, Di Biase L (2020). Impact of atrial fibrillation catheter ablation on mortality, stroke, and heart failure hospitalizations: a meta-analysis. J Cardiovasc Electrophysiol.

[29] Nattel S, Harada M (2014). Atrial remodeling and atrial fibrillation: recent advances and translational perspectives. J Am Coll Cardiol.

[30] Verma A, Natale A (2005). Should atrial fibrillation ablation be considered first-line therapy for some patients? Why atrial fibrillation ablation should be considered first-line therapy for some patients. Circulation.

[31] Chew DS, Black-Maier E, Loring Z (2020). Diagnosis-to-ablation time and recurrence of atrial fibrillation following catheter ablation: a systematic review and meta-analysis of observational studies. Circ Arrhythm Electrophysiol.

[32] Lip GYH, Ntaios G (2021). “Novel clinical concepts in thrombosis”: integrated care for stroke management-easy as ABC. Thromb Haemost.

[33] Field M, Kuduvalli M, Torella F (2021). Integrated care systems and the aortovascular hub. Thromb Haemost.

[34] Yoon M, Yang P-S, Jang E (2019). Improved population-based clinical outcomes of patients with atrial fibrillation by compliance with the simple abc (atrial fibrillation better care) pathway for integrated care management: a nationwide cohort study. Thromb Haemost.

[35] Romiti GF, Pastori D, Rivera-Caravaca JM (2021). Adherence to the “atrial fibrillation better care” pathway in patients with atrial fibrillation: impact on clinical outcomes-a systematic review and meta-analysis of 285,000 patients. Thromb Haemost.

