# Supplementary material for: Impact of early ablation of atrial fibrillation on long-term outcomes: results from phase II/III of the GLORIA-AF registry
Source: Clin Res Cardiol. 2022 Apr 29;111(9):1057–68. doi: 10.1007/s00392-022-02022-1 (PMC9424157; doi:10.1007/s00392-022-02022-1)
Supplement: Supplementary file 1 — Supplementary file1 (DOCX 371 KB) [file 392_2022_2022_MOESM1_ESM.docx]

Supplementary Materials

Impact of Early Ablation of Atrial Fibrillation on Long-Term Outcomes: Results from Phase II/III of the GLORIA-AF Registry.

Short title: Impact of early AF ablation

Wern Yew Ding^1^; Peter Calvert^1^; Dhiraj Gupta^1^; Menno V Huisman^2*^; Gregory Y. H. Lip^1,3*^; on behalf of the GLORIA-AF Investigators^4^

^1^ Liverpool Centre for Cardiovascular Science, University of Liverpool and Liverpool Heart & Chest Hospital, Liverpool, United Kingdom; ^2^ Department of Thrombosis and Hemostasis, Leiden University Medical Center, Leiden, the Netherlands; ^3^ Aalborg Thrombosis Research Unit, Department of Clinical Medicine, Aalborg University, Aalborg, Denmark; ^4^ Listed in Appendix.

[*Drs Huisman and Lip and co-Chairs of the GLORIA-AF registry and joint senior authors]

Corresponding author:

Prof Gregory Y H Lip [gregory.lip@liverpool.ac.uk](mailto:gregory.lip@liverpool.ac.uk)


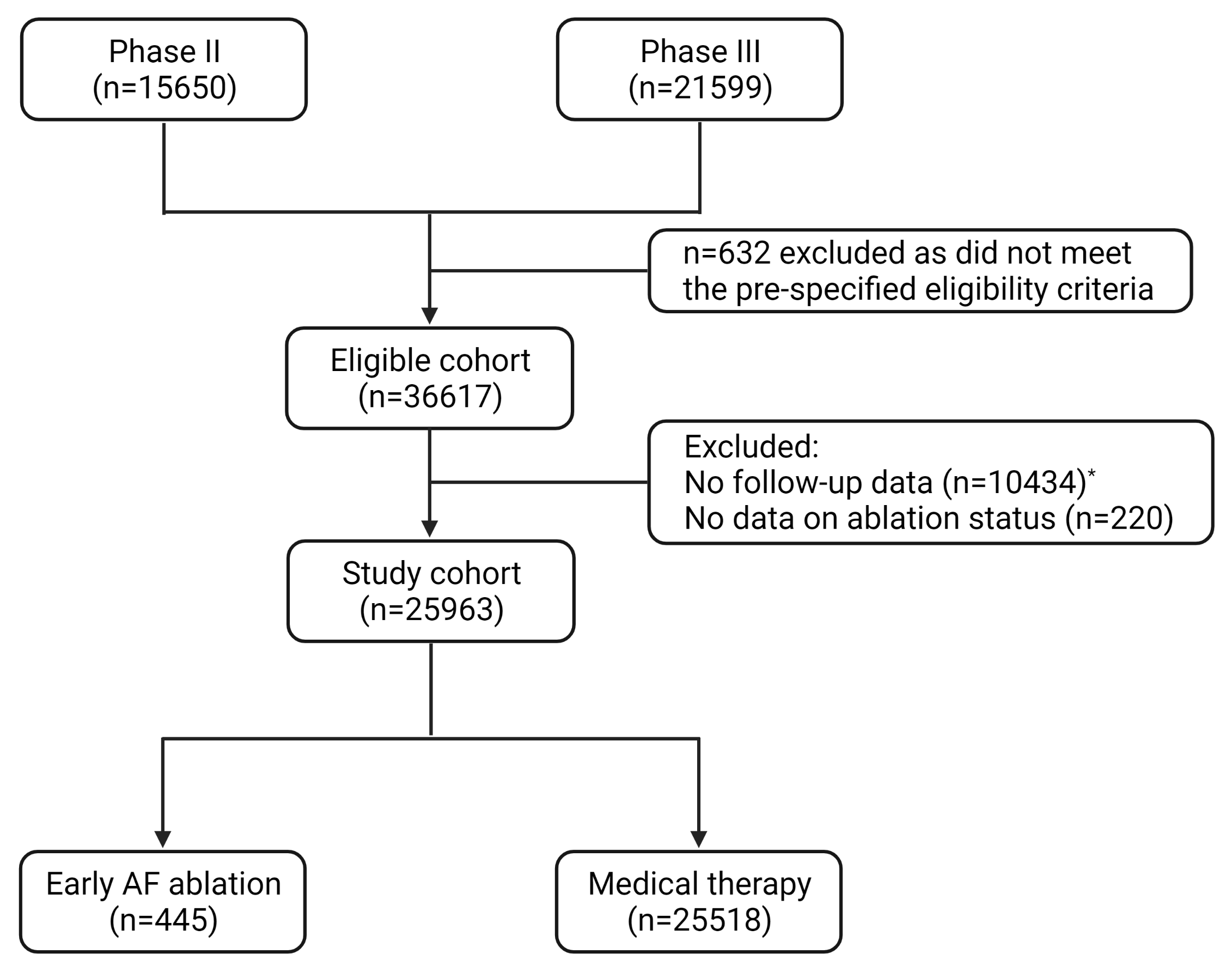


**Supplementary Figure 1**. Flow chart of patient selection.

^*^ In phase II of the GLORIA-AF registry, only patients in the dabigatran subgroup had follow-up data.

**Supplementary Table 1.** Baseline characteristics *after* propensity score matching

| **Baseline characteristics** | **Early AF ablation**  **(n = 399)** | **Medical therapy**  **(n = 399)** | **p value** | **SMD** |
| --- | --- | --- | --- | --- |
| Age (years), median (IQR) | 63 (57 - 70) | 65.0 (54.5 - 72.0) | 0.343 | 0.029 |
| Female sex, n (%) | 172 (4.1%) | 167 (41.9%) | 0.775 | 0.025 |
| BMI (kg/m^2^), median (IQR) | 26.1 (23.6 - 29.1) | 26.5 (23.6 - 29.4) | 0.470 | 0.003 |
| CrCl (mL/min), median (IQR) | 84.8 (69.0 - 107.0) | 84.6 (65.9 - 109.0) | 0.537 | 0.436 |
| AF classification, n (%) |  |  | 0.840 |  |
| Paroxysmal | 295 (73.9%) | 287 (71.9%) |  | 0.046 |
| Persistent | 102 (25.6%) | 110 (27.6%) |  | 0.046 |
| Permanent | 2 (0.5%) | 2 (0.5%) |  | 0.000 |
| Comorbidities, n (%) |  |  |  |  |
| Hypertension | 275 (68.9%) | 283 (70.9%) | 0.589 | 0.043 |
| Hypercholesterolaemia | 114 (28.6%) | 113 (28.3%) | 1.000 | 0.006 |
| Diabetes mellitus | 70 (17.5%) | 75 (18.8%) | 0.713 | 0.033 |
| Coronary artery disease | 78 (19.5%) | 82 (20.6%) | 0.791 | 0.025 |
| Prior myocardial infarction | 10 (2.5%) | 9 (2.3%) | 1.000 | 0.016 |
| Congestive heart failure | 53 (13.3%) | 54 (13.5%) | 1.000 | 0.007 |
| LVH | 46 (11.5%) | 39 (9.8%) | 0.491 | 0.055 |
| Prior thromboembolism | 43 (10.8%) | 39 (9.8%) | 0.727 | 0.032 |
| Prior bleeding | 14 (3.5%) | 21 (5.3%) | 0.300 | 0.095 |
| Peripheral artery disease | 5 (1.3%) | 2 (0.5%) | 0.451 | 0.068 |
| COPD | 11 (2.8%) | 11 (2.8%) | 1.000 | 0.000 |

AF, atrial fibrillation; BMI, body mass index; COPD, chronic obstructive pulmonary disease; CrCl, creatinine clearance; IQR, interquartile range; LVH, left ventricular hypertrophy; SMD, standardised mean difference.
